# Incorporation of doxorubicin in different polymer nanoparticles and their anticancer activity

**DOI:** 10.3762/bjnano.10.201

**Published:** 2019-10-29

**Authors:** Sebastian Pieper, Hannah Onafuye, Dennis Mulac, Jindrich Cinatl, Mark N Wass, Martin Michaelis, Klaus Langer

**Affiliations:** 1 Institute of Pharmaceutical Technology and Biopharmacy, University of Muenster, Corrensstraße 48, 48149 Muenster, Germany; 2 Industrial Biotechnology Centre and School of Biosciences, University of Kent, Canterbury CT2 7NJ, United Kingdom; 3 Institute for Medical Virology, University Hospital, Goethe-University, Paul Ehrlich-Straße 40, 60596 Frankfurt am Main, Germany

**Keywords:** cancer, doxorubicin, drug release, nanoparticles, poly(lactic-*co*-glycolic acid) (PLGA)

## Abstract

**Background:** Nanoparticles are under investigation as carrier systems for anticancer drugs. The expression of efflux transporters such as the ATP-binding cassette (ABC) transporter ABCB1 is an important resistance mechanism in therapy-refractory cancer cells. Drug encapsulation into nanoparticles has been shown to bypass efflux-mediated drug resistance, but there are also conflicting results. To investigate whether easy-to-prepare nanoparticles made of well-tolerated polymers may circumvent transporter-mediated drug efflux, we prepared poly(lactic-*co*-glycolic acid) (PLGA), polylactic acid (PLA), and PEGylated PLGA (PLGA-PEG) nanoparticles loaded with the ABCB1 substrate doxorubicin by solvent displacement and emulsion diffusion approaches and assessed their anticancer efficiency in neuroblastoma cells, including ABCB1-expressing cell lines, in comparison to doxorubicin solution.

**Results:** The resulting nanoparticles covered a size range between 73 and 246 nm. PLGA-PEG nanoparticle preparation by solvent displacement led to the smallest nanoparticles. In PLGA nanoparticles, the drug load could be optimised using solvent displacement at pH 7 reaching 53 µg doxorubicin/mg nanoparticle. These PLGA nanoparticles displayed sustained doxorubicin release kinetics compared to the more burst-like kinetics of the other preparations. In neuroblastoma cells, doxorubicin-loaded PLGA-PEG nanoparticles (presumably due to their small size) and PLGA nanoparticles prepared by solvent displacement at pH 7 (presumably due to their high drug load and superior drug release kinetics) exerted the strongest anticancer effects. However, nanoparticle-encapsulated doxorubicin did not display increased efficacy in ABCB1-expressing cells relative to doxorubicin solution.

**Conclusion:** Doxorubicin-loaded nanoparticles made by different methods from different materials displayed substantial discrepancies in their anticancer activity at the cellular level. Optimised preparation methods resulted in PLGA nanoparticles characterised by increased drug load, controlled drug release, and high anticancer efficacy. The design of drug-loaded nanoparticles with optimised anticancer activity at the cellular level is an important step in the development of improved nanoparticle preparations for anticancer therapy. Further research is required to understand under which circumstances nanoparticles can be used to overcome efflux-mediated resistance in cancer cells.

## Introduction

According to Globocan, there “were 14.1 million new cancer cases, 8.2 million cancer deaths and 32.6 million people living with cancer (within five years of diagnosis) in 2012 worldwide” [1]. Despite substantial improvements over recent decades, the prognosis for many cancer patients remains unacceptably poor. In particular, the outlook is grim for patients that are diagnosed with disseminated (metastatic) disease who cannot be successfully treated by local treatment (surgery, radiotherapy). These patients depend on systemic drug therapy. However, the therapeutic window is small, and anticancer therapies are typically associated with severe side-effects [2–3].

One strategy to develop more effective cancer therapies is to use nano-sized drug delivery systems that mediate a more specific tumour accumulation of transported drugs. Tumour targeting can be achieved via the enhanced permeability and retention (EPR) effect, which is the consequence of increased leakiness of the tumour vasculature and a lack of lymph drainage [4]. Nano-sized drug carrier systems can also prolong the circulation time of anticancer drugs, protect them from degradation, and sustain therapeutic drug concentrations due to prolonged/controlled drug release. In addition, nanoparticles can be used to administer poorly soluble agents, as demonstrated for nab-paclitaxel, a HSA nanoparticle-based paclitaxel preparation approved for the treatment of different forms of cancer [4–9].

Another important aspect of the efficacy of nanoparticles as delivery system for anticancer is their uptake and, in turn, the drug transport into cancer cells. Uptake mechanisms may differ between different types of nanoparticles, which may affect their effectiveness as carriers for anticancer drugs. Here, we prepared and directly compared the effects of doxorubicin-loaded polylactic acid (PLA) and poly(lactic-*co*-glycolic acid) (PLGA) nanoparticles in neuroblastoma cells. PLA and PLGA are well-known ingredients of FDA- and EMA-approved drugs for human use [10–11] and are easily degraded into their monomers, lactic acid and glycolic acid. Furthermore, a copolymer composed of polyethylene glycol (PEG) and PLGA (PLGA-PEG) was used for nanoparticle preparation. PEGylated (“stealth”) nanoparticles display prolonged systemic circulation time, because they avoid agglomeration, opsonisation, and phagocytosis [12].

In previous studies PLA-, PLGA-, PLA-PEG-, and PLGA-PEG-based nanometre-sized drug carriers loaded with or covalently linked to doxorubicin have been prepared by methods including emulsion diffusion, solvent displacement, micelle formation, and film rehydration followed by pH-gradient method [13–19].

The expression of ATP-binding cassette (ABC) transporters such as ABCB1 (also known as MDR1 or P-glycoprotein/P-gp), which efflux a range of anticancer drugs, is an important drug resistance mechanism in cancer cells [20–21]. Different nano-sized drug carrier systems including PLA-, PLGA-, and PEG-based preparations have been reported to bypass the transporter-mediated efflux of anticancer drugs including doxorubicin [20,22–31]. However, there are also conflicting results from studies in which encapsulation of anticancer drugs into nanoparticles did not result in increased efficacy in ABCB1-expressing cancer cells relative to drug solution [19,32–33]. Hence, systematic studies are required to better understand the prospects and limitations of nanoparticles as carriers for anticancer drugs, in particular in the context of efflux-mediated resistance.

Since nanoparticles prepared by simple methods have the highest chance of clinical translation, doxorubicin was incorporated into nanoparticles prepared from PLA, PLGA, and PLGA-PEG by emulsion diffusion or solvent displacement approaches, two well-established and comparatively simple preparation methods. The resulting nanoparticles were compared by particle diameter, polydispersity index, zeta potential, drug load, and drug release behaviour. Preliminary results on the preparation of doxorubicin-loaded PLGA nanoparticles have been previously published [34]. Selected preparations were tested for anticancer efficacy in cancer cell lines, including cell lines that express ABCB1.

## Results and Discussion

### Influence of the preparation technique on particle diameter and polydispersity index

**Figure 1:**
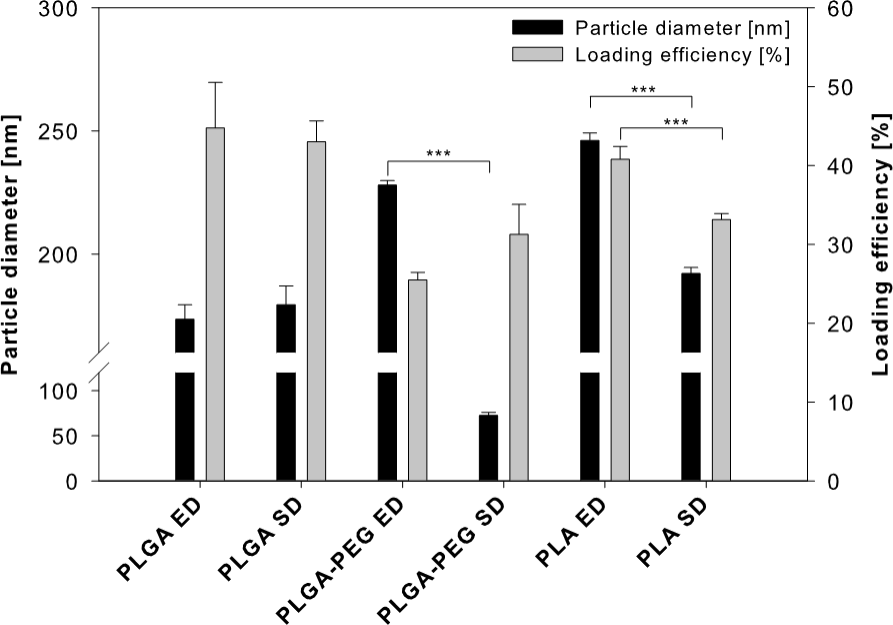
Resulting particle diameters and loading efficiencies for different nanoparticle formulations using emulsion diffusion (ED) or solvent displacement (SD) techniques (data expressed as means ± SD, *n* ≥ 3).

Nanoparticles based on poly(lactic-*co*-glycolic acid) (PLGA), a copolymer composed of polyethylene glycol (PEG) and PLGA (PLGA-PEG) and polylactic acid (PLA), respectively, were prepared in the presence of doxorubicin by either emulsion diffusion or solvent displacement technique. The resulting particle diameters are presented in Figure 1.

Emulsion diffusion (173.5 ± 5.9 nm) and solvent displacement (179.4 ± 7.6 nm) resulted in PLGA nanoparticles with similar diameters. In contrast, solvent displacement resulted in PLGA-PEG nanoparticles of 72.6 ± 3.3 nm whereas emulsion diffusion resulted in PLGA-PEG nanoparticles of 222.6 ± 3.1 nm. In accordance, solvent displacement using the stabiliser PVA at concentrations between 2% and 4% (w/v) and controlled injection at mild stirring had previously been shown to produce PLGA-PEG nanoparticles with a diameter below 100 nm [35–37]. The hydrophilic PEG chains may sterically stabilise the nanoparticles by reducing PLGA aggregation during nanoparticle formation resulting in smaller particle diameters [38].

**Figure 2:**
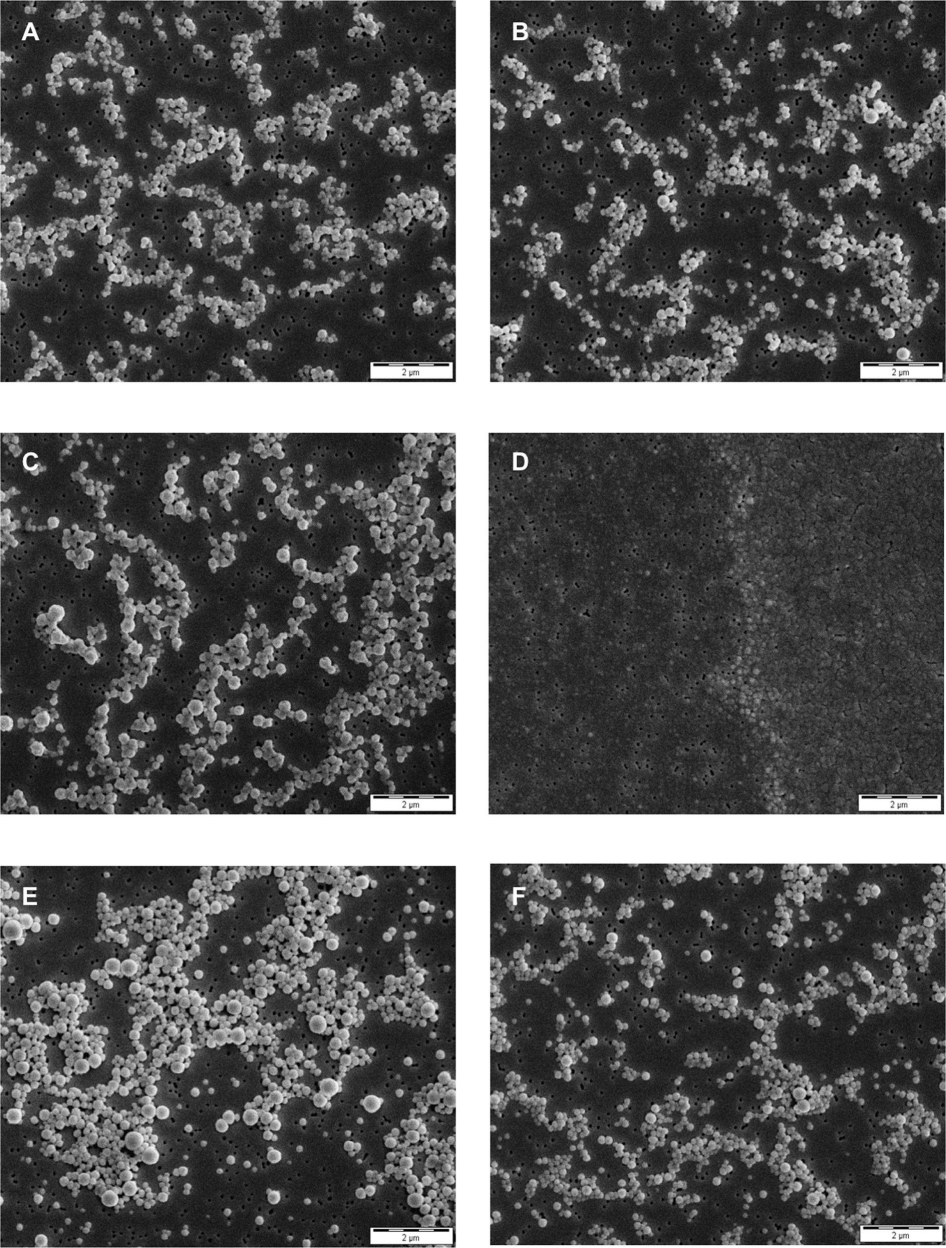
SEM images of nanoparticles using emulsion diffusion (ED) or solvent displacement (SD) preparation technique. (A) PLGA nanoparticles ED, (B) PLGA nanoparticles SD, (C) PLGA-PEG nanoparticles ED, (D) PLGA-PEG nanoparticles SD, (E) PLA nanoparticles ED, (F) PLA nanoparticles SD. Images were taken at 10,000× magnification.

Emulsion diffusion resulted in PLA nanoparticles of 246.2 ± 2.9 nm and solvent displacement in PLA nanoparticles of 192.1 ± 2.5 nm. The detailed reason for this is not clear, but in the case of the emulsion diffusion technique the resulting particle size is mainly influenced by the droplet size during the initial emulsification step. In principle, PLA nanoparticles can be prepared at a range of sizes that is determined by parameters including the preparation method, the exact polymer used, and the encapsulated drug [39–42]. Optimisation is possible [39,42] but was not subject of this study focused on the comparison of different nanoparticle systems prepared by simple methods. Polydispersity indices smaller than 0.1 indicated a monodisperse size distribution for all nanoparticle preparations. Monodispersity and particle diameters were confirmed by scanning electron microscopy (SEM) images (Figure 2).

### Influence of the preparation technique on loading efficiency and drug release

Loading efficiencies ranging from 25.5 ± 1.0% to 44.8 ± 5.8% of the applied doxorubicin were detected in the different nanoparticle preparations as shown in Figure 1, resulting in drug loads between 2.6 ± 0.2 µg doxorubicin/mg nanoparticle and 6.7 ± 0.3 µg doxorubicin/mg nanoparticle (Table 1).

**Table 1:** Nanoparticle (NP) yield and doxorubicin (Dox) drug load results for nanoparticles prepared by either emulsion diffusion (ED) or solvent displacement (SD) technique (data expressed as means ± SD, *n* ≥ 3).

NP system	NP yield [mg NP/mL]	NP yield [%]	drug load [µg Dox/mg NP]

PLGA ED	3.3 ± 0.4	66.8 ± 7.2	6.7 ± 0.3
PLGA SD	8.5 ± 0.4	70.4 ± 3.0	5.1 ± 0.2
PLGA-PEG ED	4.2 ± 0.1	84.4 ± 1.8	3.0 ± 0.2
PLGA-PEG SD	7.6 ± 0.9	63.6 ± 7.4	4.1 ± 0.6
PLA ED	8.0 ± 1.0	79.6 ± 9.8	2.6 ± 0.2
PLA SD	5.3 ± 0.2	44.1 ± 1.8	6.3 ± 0.1

In the case of PLGA and PLGA-PEG nanoparticles, emulsion diffusion and solvent displacement resulted in nanoparticles with a similar drug load. In PLA nanoparticles, there was a substantial difference between the techniques (solvent displacement: 6.3 ± 0.1 µg doxorubicin/mg nanoparticle, emulsion diffusion: 2.6 ± 0.2 µg doxorubicin/mg nanoparticle) (Table 1). The reasons underlying this difference are not clear, but emulsion diffusion has been considered of limited efficacy for the encapsulation of hydrophilic drugs [43–44].

**Figure 3:**
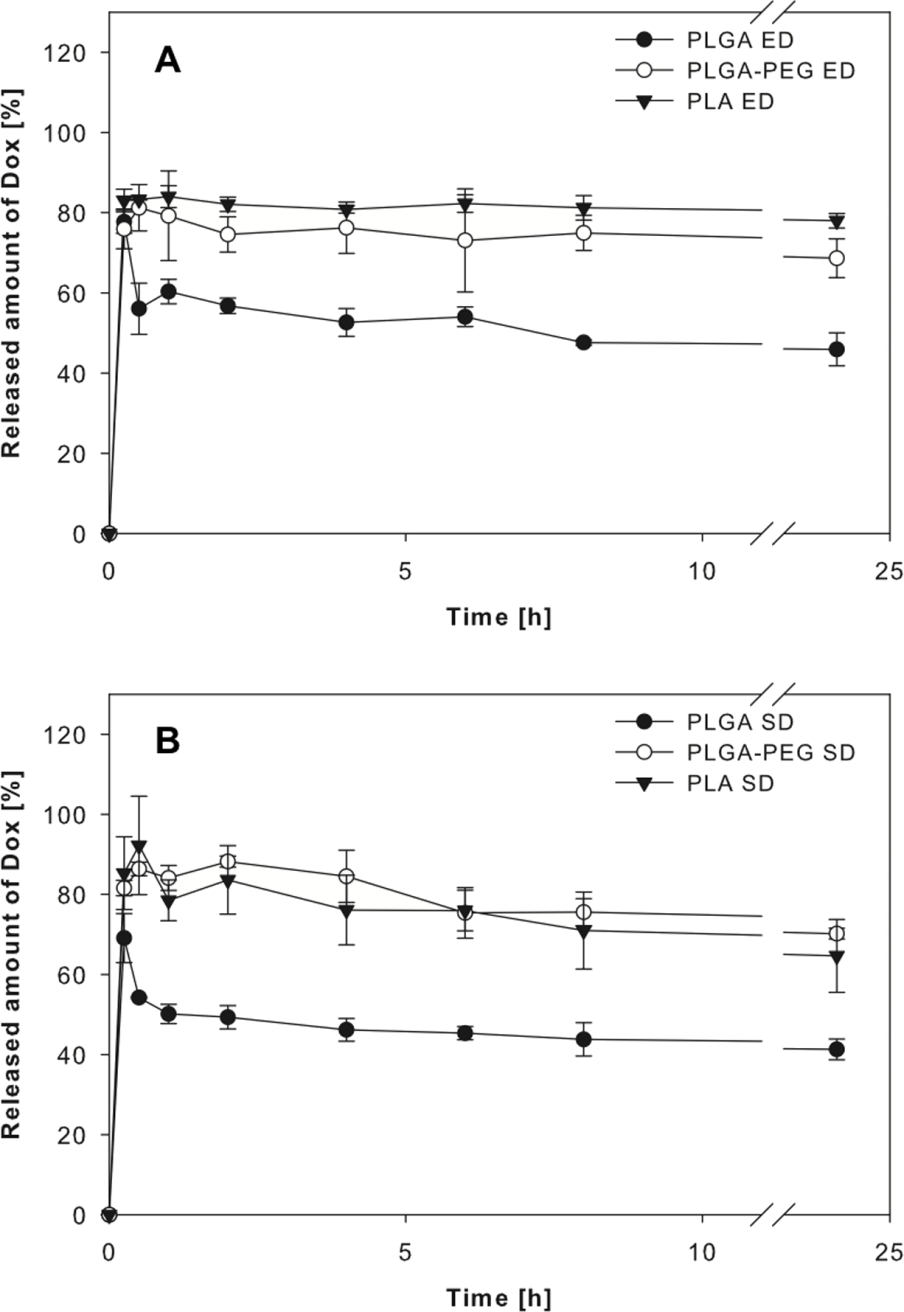
Doxorubicin (Dox) release profiles over 24 h for all nanoparticle systems using (A) emulsion diffusion (ED) or (B) solvent displacement (SD) preparation technique (data expressed as means ± SD, *n* = 3).

All nanoparticles displayed a similar drug release behaviour characterised by an initial burst release (Figure 3), which is in accordance to previous studies and may be caused by processes including the release of drug adsorbed to the nanoparticles and/or rapid drug diffusion through the particle matrix [15,39,45–47].

PEGylated polymers may result in a more porous particle structure, which is caused by aqueous channels created by PEG chains and anticipated to further increase the initial burst release [48]. However, the burst release was not increased substantially further using PLGA-PEG nanoparticles. A slight drop in the doxorubicin concentration was noticeable in the medium of the PLGA nanoparticles. This may be the consequence of doxorubicin adsorption to BSA [49], which was added to simulate the presence of plasma proteins, in combination with a slower post-burst doxorubicin release compared to the other nanoparticle systems. Such a burst release should be avoided, because it may result in drug release shortly after i.v. application before the nanoparticles reach the desired site of drug action, e.g., the tumour tissue [50].

To optimise the loading efficiency and drug release kinetics of PLGA nanoparticles the pH value of the stabiliser solution used during nanoparticle preparation was increased to 7. At this pH value, doxorubicin exists in the more lipophilic deprotonated form [51]. The use of PVA solution at pH 7 had no influence on the nanoparticle characteristics such as particle diameter, PDI, and zeta potential (Table 2).

**Table 2:** Resulting particle diameter, polydispersity index (PDI), and zeta potential (ZP) for PLGA nanoparticles prepared by an unmodified PVA solution and a PVA solution adjusted to pH 7 (data expressed as means ± SD, *n* = 3).

PVA solution	diameter [nm]	PDI	ZP [mV]

without pH adjustment	177.9 ± 1.0	0.039 ± 0.031	−41.6 ± 2.0
pH 7	174.1 ± 2.8	0.057 ± 0.030	−43.8 ± 3.7

**Figure 4:**
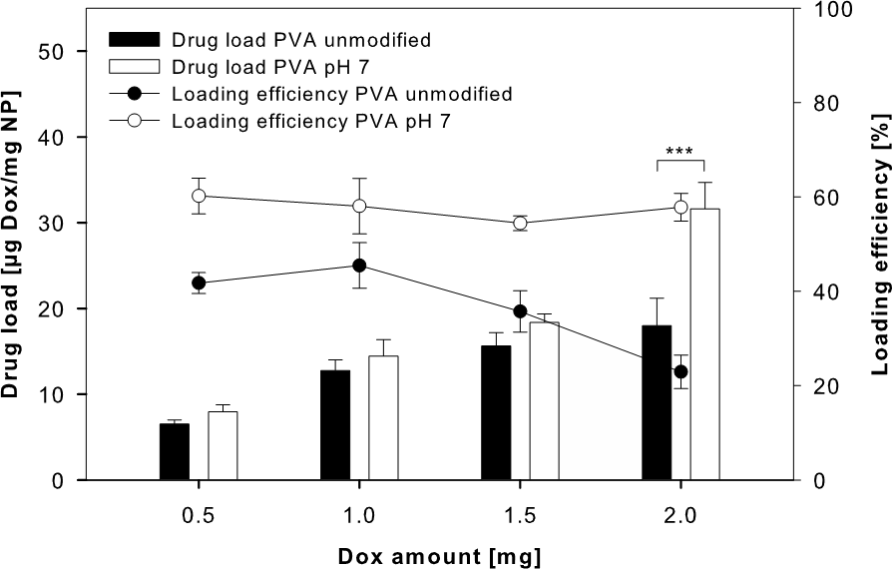
Doxorubicin (Dox) drug load and loading efficiency for PLGA nanoparticles (NPs) prepared using a PVA solution without pH adjustment and a PVA solution adjusted to pH 7 (data expressed as means ± SD, *n* = 3).

However, loading efficiency and drug load increased. The drug load raised from 6.7 ± 0.3 µg doxorubicin/mg nanoparticle (44.8 ± 5.8% loading efficiency) without pH adjustment to 7.9 ± 0.8 µg doxorubicin/mg nanoparticle (60.2 ± 3.8% loading efficiency) at pH 7. By increasing the amount of doxorubicin to 2 mg, the drug load of PLGA nanoparticles could be further enhanced (non-adjusted pH: 18.0 ± 3.2 µg doxorubicin/mg nanoparticle; pH 7: 31.6 ± 3.1 µg doxorubicin/mg nanoparticle, respectively) (Figure 4).

**Figure 5:**
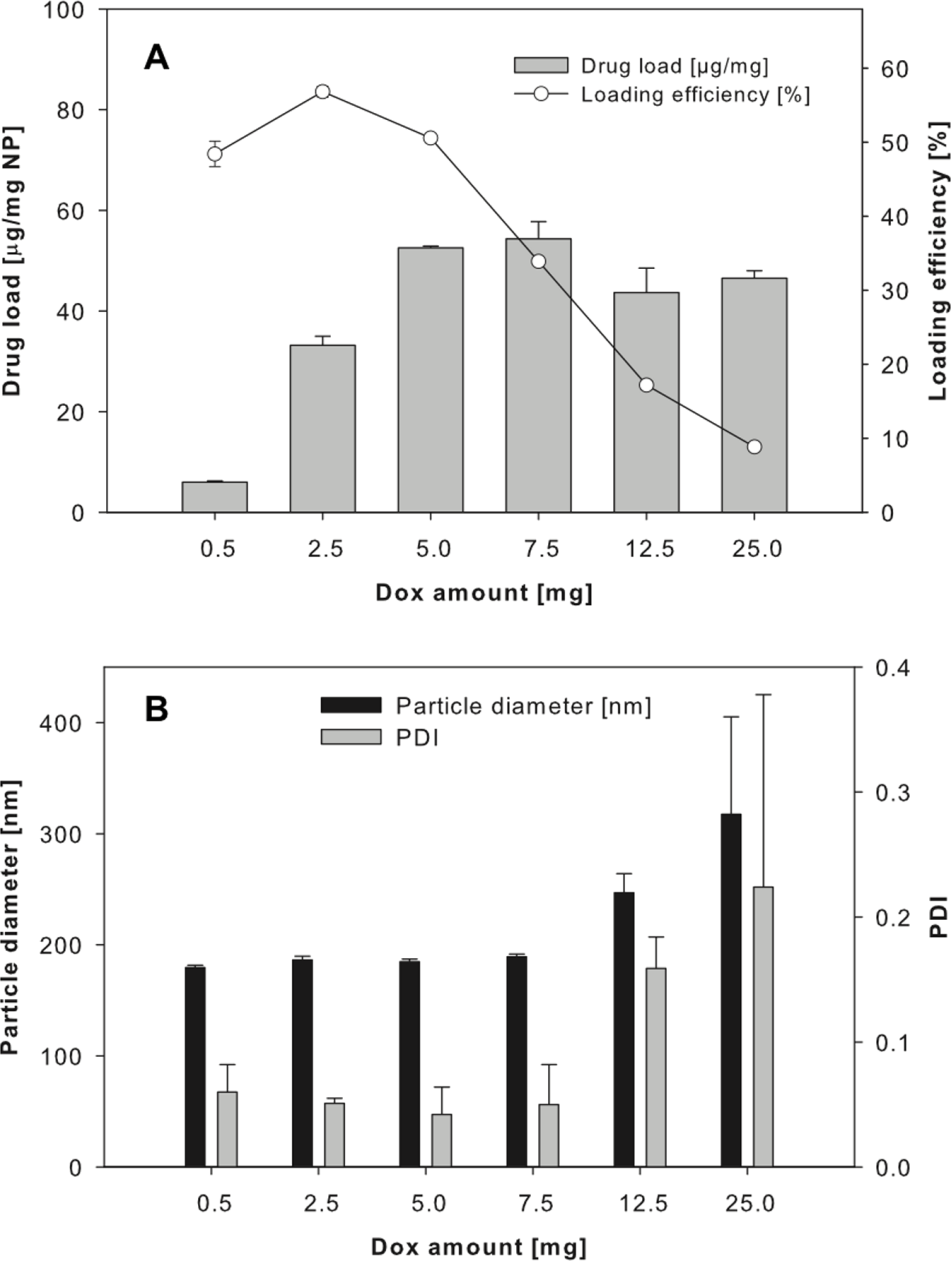
(A) Drug load and loading efficiencies as well as (B) particle diameter and PDI for different amounts of doxorubicin (Dox) used for the preparation of PLGA nanoparticles by emulsion diffusion technique (data expressed as means ± SD, *n* = 3).

Different amounts of doxorubicin did not change the loading efficiency at pH 7. Using aqueous solutions instead of methanol, we increased the doxorubicin amount during preparation to 5 mg and 7.5 mg per 50 mg PLGA. While 5 mg resulted in an increase of the drug load to 52.5 ± 0.4 µg doxorubicin/mg nanoparticle, 7.5 mg doxorubicin did not result in a significant further increase (54.4 ± 3.4 µg doxorubicin/mg nanoparticle) (Figure 5A).

This was an improvement in drug load compared to a nanoparticle preparation in the presence of 2 mg doxorubicin. However, a further increase of doxorubicin resulted in unstable nanoparticle systems, as indicated by increasing particle diameter and polydispersity index (Figure 5B). The loading efficiency for PLGA nanoparticles prepared at pH 7 with 5 mg doxorubicin was higher than this for nanoparticles manufactured with 7.5 mg doxorubicin (50.6 ± 0.6% and 33.9 ± 0.5%, respectively). These loading efficiencies are in the range of those described for similar preparations, although higher drug loads have been described when using alternative PLGA-based formulations such as nanoparticles or micelles with doxorubicin covalently bound to the polymer, nanoparticles produced by nanoprecipitation, micelles based on multi-arm star-shaped PLGA–PEG block copolymers, or nanopolymersomes [14–18].

**Figure 6:**
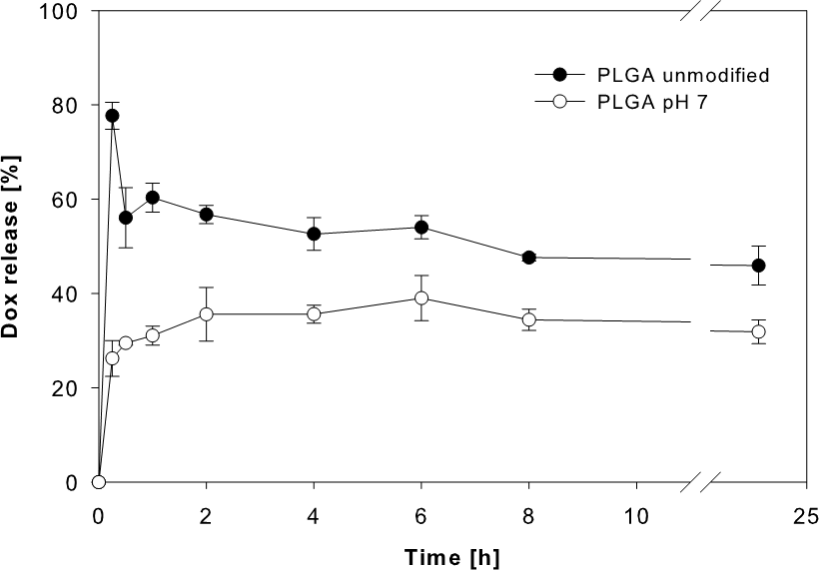
Release profiles of doxorubicin from PLGA nanoparticles prepared using an unmodified PVA solution and a PVA solution adjusted to pH 7 (data expressed as means ± SD, *n* = 3).

In addition, PLGA nanoparticles prepared at pH 7 displayed a more controlled and sustained doxorubicin release than PLGA nanoparticles prepared without pH adjustment (Figure 6). Hence, PLGA nanoparticles prepared at pH 7 with 5 mg doxorubicin were selected for cell culture experiments.

The different release kinetics from PLGA nanoparticles prepared at pH 7, may be attributed to the higher lipophilicity of doxorubicin at this pH value and, in turn, a stronger incorporation into the lipophilic PLGA nanoparticle matrix. This explanation is consistent with data showing that PLGA nanoparticle degradation is unlikely to occur in a 24 h timeframe [50,52]. More sustained release patterns have been shown to be achievable by alternative nanoparticle approaches based on PLGA such as nanoparticles or micelles with doxorubicin covalently bound to the polymer, nanoparticles produced by nanoprecipitation, micelles based on multi-arm star-shaped PLGA–PEG block copolymers, or nanopolymersomes [14–18].

### Nanoparticle efficacy in cell culture

**Figure 7:**
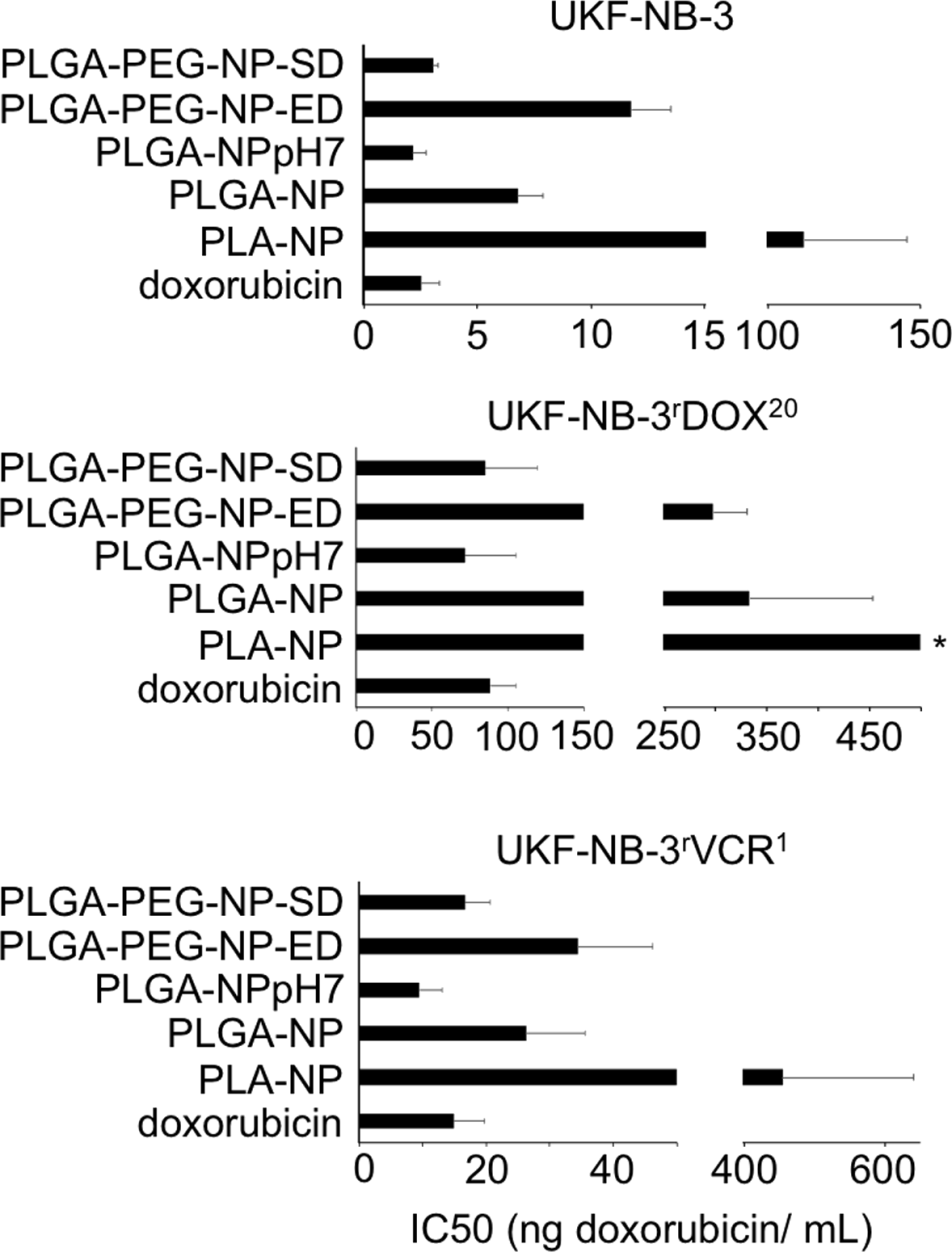
Doxorubicin concentrations that reduce neuroblastoma cell viability by 50% (IC50) when administered encapsulated into different nanoparticle preparations (PLA-NP, PLA nanoparticles prepared by solvent displacement; PLGA-NP, PLGA nanoparticles prepared by solvent displacement at a non-adjusted pH value; PLGA-NPpH7, PLGA nanoparticles prepared by solvent displacement at pH 7; PLGA-PEG-ED, PLGA-PEG nanoparticles prepared by emulsion diffusion; PLGA-PEG-SD, PLGA-PEG nanoparticles prepared by solvent displacement) compared to doxorubicin solution (doxorubicin). Unloaded nanoparticles did not affect cell viability in the tested concentration range. *IC50 > 500 ng/mL.

Finally, the effects of doxorubicin-loaded PLA nanoparticles prepared by solvent displacement (because they were smaller and the drug load was higher compared to those prepared by emulsion diffusion), PLGA nanoparticles prepared by solvent displacement at a non-adjusted pH value and at pH 7, and PLGA-PEG nanoparticles prepared by emulsion diffusion and solvent displacement were tested for their effects on the viability of the neuroblastoma cell line UKF-NB-3, its doxorubicin-adapted sub-line UKF-NB-3^r^DOX^20^, and its vincristine-resistant sub-line UKF-NB-3^r^VCR^1^. In all three cell lines, PLA nanoparticles, PLGA nanoparticles prepared by solvent displacement at a non-adjusted pH value, and PLGA-PEG nanoparticles prepared by emulsion diffusion displayed reduced efficacy compared to doxorubicin solution (Figure 7).

In contrast, PLGA nanoparticles prepared by solvent displacement at pH 7 and PLGA-PEG nanoparticles prepared by solvent displacement were similarly active as free doxorubicin (Figure 7). The corresponding empty nanoparticles did not affect cell viability in the tested concentrations.

The main difference between the doxorubicin-loaded PLGA-PEG nanoparticles prepared by solvent displacement and the other preparations is the size. It is the only preparation in which nanoparticles have a size clearly smaller than 100 nm (72.6 ± 3.3 nm, Figure 1). This might indicate that the cellular uptake of smaller nanoparticles is higher than that of larger nanoparticles, which is coherent with previous findings showing that cellular uptake of nanoparticles decreases with an increase of size [53]. PLGA nanoparticles prepared by solvent displacement at pH 7 displayed the highest drug load. Hence, their superior effects may be explained by an increased drug transport per nanoparticle into cancer cells.

**Figure 8:**
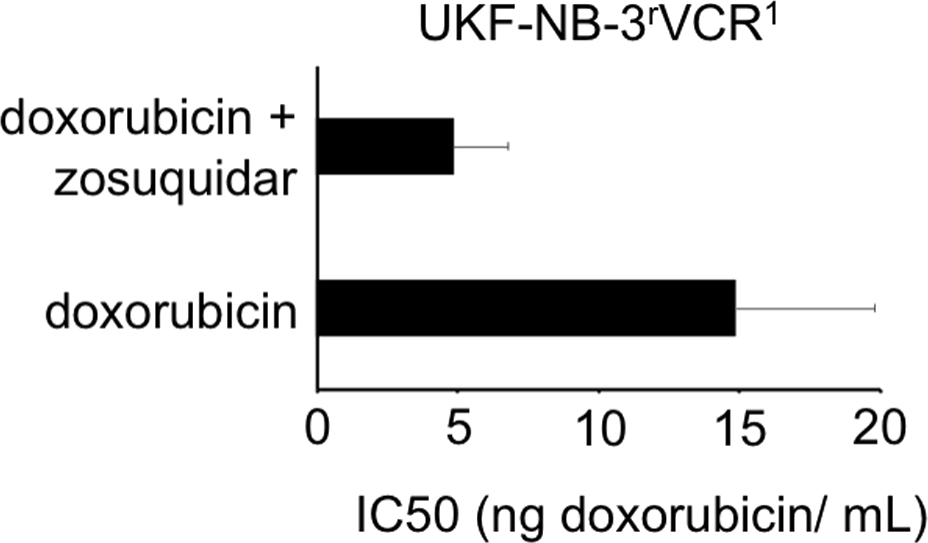
Doxorubicin concentrations that reduce UKF-NB-3^r^VCR^1^ viability by 50% (IC50) in the absence or presence of the ABCB1 inhibitor zosuquidar (1 µM). Zosuquidar did not affect cell viability when administered alone.

Nano-sized drug carriers have been shown to bypass efflux-mediated drug resistance [25]. This included various nanoparticle and liposome formulations of the ABCB1 substrate doxorubicin that were shown to modify the cellular uptake and intracellular distribution of doxorubicin resulting in enhanced effects against ABCB1-expressing cancer cells, when compared to free doxorubicin in solution [26–31]. The doxorubicin-adapted UKF-NB-3 sub-line UKF-NB-3^r^DOX^20^ is characterised by high ABCB1 expression [54]. In addition, the vincristine-resistant UKF-NB-3 sub-line UKF-NB-3^r^VCR^1^ displays cross-resistance to doxorubicin and becomes sensitised to doxorubicin by the specific ABCB1 inhibitor zosuquidar (Figure 8). This indicates that drug resistance is at least in part mediated by ABCB1 in this cell line. However, free doxorubicin solution and doxorubicin bound to PLGA-PEG nanoparticles prepared by solvent displacement or PLGA nanoparticles prepared by solvent displacement at pH 7 displayed similar efficacy in UKF-NB-3^r^DOX^20^ and UKF-NB-3^r^VCR^1^ cells (Figure 7). Hence, these drug carrier systems are not able to overcome transporter-mediated drug resistance. One reason for this may be that the doxorubicin burst release kinetics observed for these nanoparticles do not enable a sufficient bypassing of transporter-mediated drug efflux. However, PLGA nanoparticles prepared by solvent displacement at pH 7 did not display improved efficacy in ABCB1-expressing cells despite improved drug release kinetics. Possibly, other PLGA-based preparations, which display more sustained drug release, such as nanoparticles or micelles with doxorubicin covalently bound to the polymer, nanoparticles produced by nanoprecipitation, micelles based on multi-arm star-shaped PLGA–PEG block copolymers, or nanopolymersomes [14–18] may overcome such limitations.

## Conclusion

In this study, we synthesised a range of doxorubicin-loaded PLA- and PLGA-based nanoparticle systems using emulsion diffusion and solvent displacement approaches. Our results show that particle size, loading efficiency, and drug release kinetics can be controlled by the production procedure. Testing of the nanoparticle preparations in the neuroblastoma cell line UKF-NB-3 and its sub-lines with acquired resistance to doxorubicin or vincristine indicated that smaller nanoparticles and a high drug load result in nanoparticle preparations that have a similar efficacy at the cellular level as doxorubicin solution. In particular, doxorubicin-loaded PLGA-PEG nanoparticles prepared by solvent displacement, which displayed the smallest diameter, and PLGA nanoparticles prepared by solvent displacement at pH 7, which displayed the highest drug load, exerted the most pronounced anticancer effects, which were comparable to doxorubicin solution. Since nanoparticle preparations are known to have the capacity to improve the in vivo activity of anticancer drugs by tumour targeting through the EPR effect, this is an important step in the development of improved nanoparticle preparations. However, the investigated nanoparticle preparations did not circumvent transporter-mediated drug efflux. Hence, more research is required to identify drug carrier systems that reliably bypass efflux-mediated drug resistance.

## Experimental

### Reagents

PLGA (Resomer^®^ RG502H), PLA (Resomer^®^ R203H), and PLGA-PEG (Resomer^®^ RGP d 50155) were obtained from Evonik Industries AG (Essen, Germany). Ethyl acetate, dichloromethane, and methanol were purchased from VWR International GmbH (Darmstadt, Germany). Acetone, acetonitrile and dimethyl sulfoxide (DMSO) were obtained from Carl Roth GmbH (Karlsruhe, Germany). Poly(vinyl alcohol) (PVA, 30,000–70,000 Da), bovine serum albumin (BSA), HSA, and glutaraldehyde were obtained from Sigma-Aldrich Chemie GmbH (Karlsruhe, Germany). Dulbecco's Phosphate buffered saline (PBS) was purchased from Biochrom GmbH (Berlin, Germany). Doxorubicin was obtained from LGC Standards GmbH (Wesel, Germany). All chemicals were of analytical grade and used as received.

### Nanoparticle preparation via emulsion diffusion

PLA and PLGA nanoparticles were prepared by a previously described emulsion diffusion technique [35,55]. PLA, PLGA, or PLGA-PEG were dissolved in organic solvents (Table 3) and 200 µL of a methanolic doxorubicin solution (2.5 mg/mL) was added.

**Table 3:** Preparation parameters for nanoparticles based on different polymers using emulsion diffusion technique.

polymer	amount of polymer	organic solvent	homogenisation

PLGA	50 mg	2.5 mL ethyl acetate	15,000 rpm for 5 min
PLA	100 mg	2.0 mL dicholoromethane	18,000 rpm for 15 min
PLGA-PEG	50 mg	2.5 mL ethyl acetate	15,000 rpm for 5 min

This solution was then poured into 5 mL (1%, m/v) PVA solution and afterwards homogenized with an Ultra Turrax (IKA-Werke, Staufen, Germany) as indicated in Table 3. Subsequently this pre-emulsion was mixed with another 5 mL (1%, m/v) PVA solution. After stirring overnight, the resultant nanoparticles were purified three times by centrifugation at 21,000*g* for 15 min (Eppendorf Centrifuge 5430 R, Eppendorf, Hamburg, Germany) and re-dispersion in purified water.

After the final purification step, an aliquot of the nanoparticle suspension was centrifuged and the resulting pellet was dissolved in 1 mL DMSO in order to measure the entrapped amount of doxorubicin by HPLC (see below).

In order to increase the drug load for PLGA nanoparticles different volumes of the methanolic doxorubicin solution (2.5 mg/mL) were used corresponding to 1.0, 1.5, and 2.0 mg total doxorubicin. For a further increase in drug load different aqueous doxorubicin solutions (ranging from 10.0 to 50.0 mg/mL) were used to achieve total doxorubicin amounts of 0.5, 2.5, 5.0, 7.5, 12.5, and 25.0 mg. Here, the PLGA solution in ethyl acetate was homogenized with the aqueous doxorubicin solution and 5 mL (1%, m/v) PVA solution to achieve the pre-emulsion. In all experiments to increase the drug load the amount of the polymer was kept constant at 50 mg. To prepare doxorubicin-loaded nanoparticles at a defined pH value of 7, a PVA solution (1%, m/v) in phosphate buffer (15.6 mg/mL NaH_2_PO_4_·2H_2_O; pH adjusted to pH 7 with NaOH) was used.

### Nanoparticle preparation via solvent displacement

Nanoparticle preparation via solvent displacement was performed modified after Murakami et al. [13] as previously described by Pieper and Langer [34]. 60 mg polymer were dissolved in 2 mL acetone and combined with 200 µL methanolic doxorubicin solution (2.5 mg/mL). This mixture was injected into 4 mL 2% (m/v) PVA solution to produce PLGA and PLGA-PEG nanoparticles or into 4 mL 1% (m/v) PVA solution to produce PLA nanoparticles. After stirring overnight at 550 rpm and evaporation of the organic solvent, PLA and PLGA nanoparticles were purified three times by centrifugation at 21,000 g for 15 min and re-dispersion in purified water. PLGA-PEG nanoparticles were purified three times by centrifugation at 30,000*g* for 60 min and re-dispersion in purified water.

### Determination of particle size, size distribution and zeta potential

Average particle size and the polydispersity were measured by photon correlation spectroscopy (PCS) using a Malvern zetasizer nano (Malvern Instruments, Herrenberg, Germany). The resulting particle suspensions were diluted 1:100 with purified water and measured at a temperature of 22 °C using a backscattering angle of 173°. The zeta potential was determined with the same instrument and the same diluted nanoparticle suspension by laser Doppler microelectrophoresis.

### Scanning electron microscopy (SEM)

For scanning electron microscopy (SEM), the particle suspensions were diluted with purified water to 0.25 mg/mL. The suspension was dripped on a filter (MF-Millipore™ membrane filter VSWP, 0.1 µm) and dried for 24 h in a desiccator. Afterwards, the membranes were sputtered with gold under argon atmosphere (SCD 040, BAL-TEC, Balzers, Liechtenstein). The SEM pictures were received at an accelerating voltage of 10,000 V and a working distance of 10 mm (CamScan CS4, Cambridge Scanning Company, Cambridge, UK).

### Doxorubicin quantification via HPLC-UV

The amount of doxorubicin that had been incorporated into the nanoparticles was determined by HPLC-UV (HPLC 1200 series, Agilent Technologies GmbH, Böblingen, Germany) using a LiChroCART 250 × 4 mm LiChrospher 100 RP 18 column (Merck KGaA, Darmstadt, Germany). The mobile phase was a mixture of water and acetonitrile (70:30) containing 0.1% trifluoroacetic acid [56]. In order to obtain symmetric peaks a gradient was used. In the first 6 min the percentage of water was reduced from 70% to 50%. Subsequently within 2 min the amount of water was further decreased to 20% and then within another 2 min increased again to 70%. These conditions were hold for a final 5 min resulting in a total runtime of 15 min. While using a flow rate of 0.8 mL/min, an elution time for doxorubicin of *t* = 7.5 min was achieved. The detection of doxorubicin was performed at a wavelength of 485 nm [57].

### In vitro drug release studies

To study drug release in vitro, a nanoparticle suspension of 1 mg nanoparticles in 1 mL of PBS containing 5% (m/v) bovine serum albumin (BSA) was shaken at 37 °C with 500 rpm. Nanoparticle suspensions were centrifuged (30,000*g*, 15 min) after 0, 0.5, 1, 2, 4, 6, 8, and 24 h, and an aliquot (250 µL) of the supernatant was diluted with 750 µL ethanol (96%, v/v) in order to precipitate BSA. After a second centrifugation step (30,000*g*, 10 min) the supernatant was analysed for the amount of released doxorubicin by HPLC as mentioned above. Additionally, the resulting pellet was dissolved in DMSO in order to calculate doxorubicin recovery.

### Cell culture

The MYCN-amplified neuroblastoma cell line UKF-NB-3 was established from stage-4 neuroblastoma patients [54]. UKF-NB-3 sub-lines adapted to growth in the presence of doxorubicin 20 ng/mL (UKF-NB-3^r^DOX^20^) [54] or vincristine 1 ng/mL (UKF-NB-3^r^VCR^1^) were established by continuous exposure to step-wise increasing drug concentrations as previously described [54,58] and derived from the Resistant Cancer Cell Line (RCCL) collection (https://research.kent.ac.uk/ibc/the-resistant-cancer-cell-line-rccl-collection/).

All cells were propagated in Iscove’s modified Dulbecco’s medium (IMDM) supplemented with 10% foetal calf serum, 100 U/mL penicillin and 100 mg/mL streptomycin at 37 °C. The drug-adapted sub-lines were continuously cultured in the presence of the indicated drug concentrations. Cells were routinely tested for mycoplasma contamination and authenticated by short tandem repeat profiling.

### Cell viability assay

Cell viability was determined by 3-(4,5-dimethylthiazol-2-yl)-2,5-diphenyltetrazolium bromide (MTT) assay modified after Mosmann [59], as previously described [55]. 2 × 10^4^ cells suspended in 100 µL cell culture medium were plated per well in 96-well plates and incubated in the presence of various concentrations of drug or drug preparations for 120 h. Where indicated, the ABCB1 inhibitor zosuquidar was added at a fixed concentration of 1 µM. Then, 25 µL of MTT solution (1 mg/mL (w/v) in PBS) were added per well, and the plates were incubated at 37 °C for an additional 4 h. After this, cells were lysed using 200 µL of a buffer containing 20% (w/v) sodium dodecylsulfate and 50% (v/v) *N*,*N*-dimethylformamide (pH 4.7) at 37 °C for 4 h. Absorbance was determined at 570 nm for each well using a 96-well multiscanner. After subtracting of the background absorption, the results are expressed as percentage viability relative to untreated control cultures. Drug concentrations that inhibited cell viability by 50% (IC50) were determined using CalcuSyn (Biosoft, Cambridge, UK).

### Statistical methods

All experiments of nanoparticle preparation and characterisation were performed at least three times. The results are shown as average value with standard deviation. Significance tests were conducted with Sigma Plot 12.5 (Systat Software GmbH, Erkrath, Germany), using a one-way ANOVA test with the Holm–Sidak post test. Significance levels were depicted as * for *p* ≤ 0.05, ** for *p* ≤ 0.01, and *** for *p* ≤ 0.001.
